# Radiative cooling technology with artificial intelligence

**DOI:** 10.1016/j.isci.2024.111325

**Published:** 2024-11-05

**Authors:** Yeongju Jung, Seung Hwan Ko

**Affiliations:** 1 Applied Nano and Thermal Science Lab, Department of Mechanical Engineering, Seoul National University, 1 Gwanak-ro, Gwanak-gu, Seoul 08826, South Korea; 2 Institute of Advanced Machinery and Design (SNU-IAMD), Seoul National University, Gwanak-ro, Gwanak-gu, Seoul 08826, South Korea; 3 Institute of Engineering Research / Institute of Advanced Machines and Design, Seoul National University, Seoul 08826, South Korea

**Keywords:** Artificial intelligence, Energy management, Engineering

## Abstract

As sustainable thermal management becomes a global priority, the development of radiative cooling (RC) technology has recently emerged as a promising solution. Simultaneously, recent advent of artificial intelligence (AI) offers the potential to revolutionize current research in sustainable cooling strategies. This article discusses the advancement of radiative cooling technology through the integration of AI, tackling the challenging issues arising from the conventional approach and offering strategic solutions to address global issues. AI, capable of mimicking or exceeding human capabilities through various algorithms, enables the efficient optimization of RC structures. Moreover, integrating AI with advanced RC technologies, which have the potential to surpass traditional RC configurations and applications but are still in the early stages, can further accelerate progress in the field of RC. Hence, AI-driven RC technologies will contribute to addressing the increasingly prevalent environmental challenges, further being a leading solution for next-generation sustainable thermal managements as these technologies continue to mature.

## Graphical abstract

**Figure:**
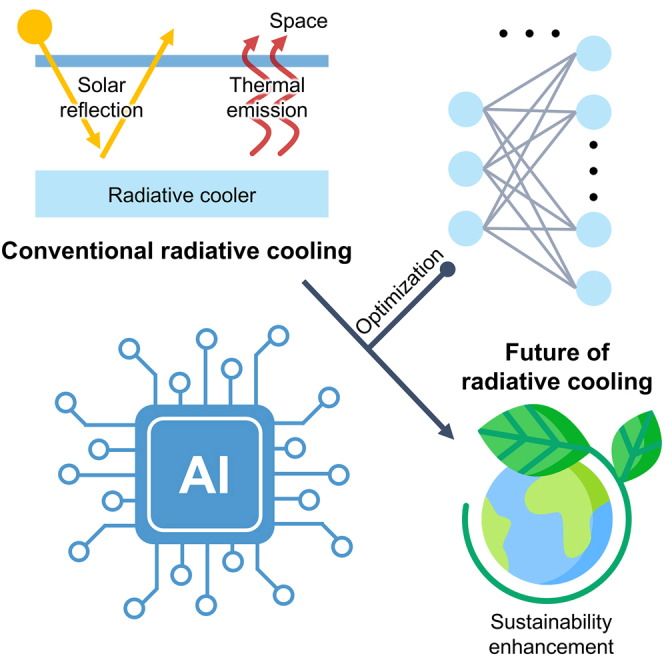

## Introduction

The energy consumption for cooling in buildings and vehicles is substantial due to global warming and urbanization.^1^^,^^2^^,^^3^ The traditional active cooling systems further contribute to the acceleration of global warming due to the release of greenhouse gases such as carbon dioxide, thereby significantly creating a harmful feedback cycle that increases the demand for air conditioning.^4^^,^^5^ To address the global challenges, various sustainable energy harvesting technologies have been developed by supplying the energy needed for cooling systems. However, they do not address the fundamental issues related to environmental pollution. Thus, the energy harvesting technologies alone do not constitute a comprehensive solution to the inherent problems of cooling technologies.

**Figure 1:**
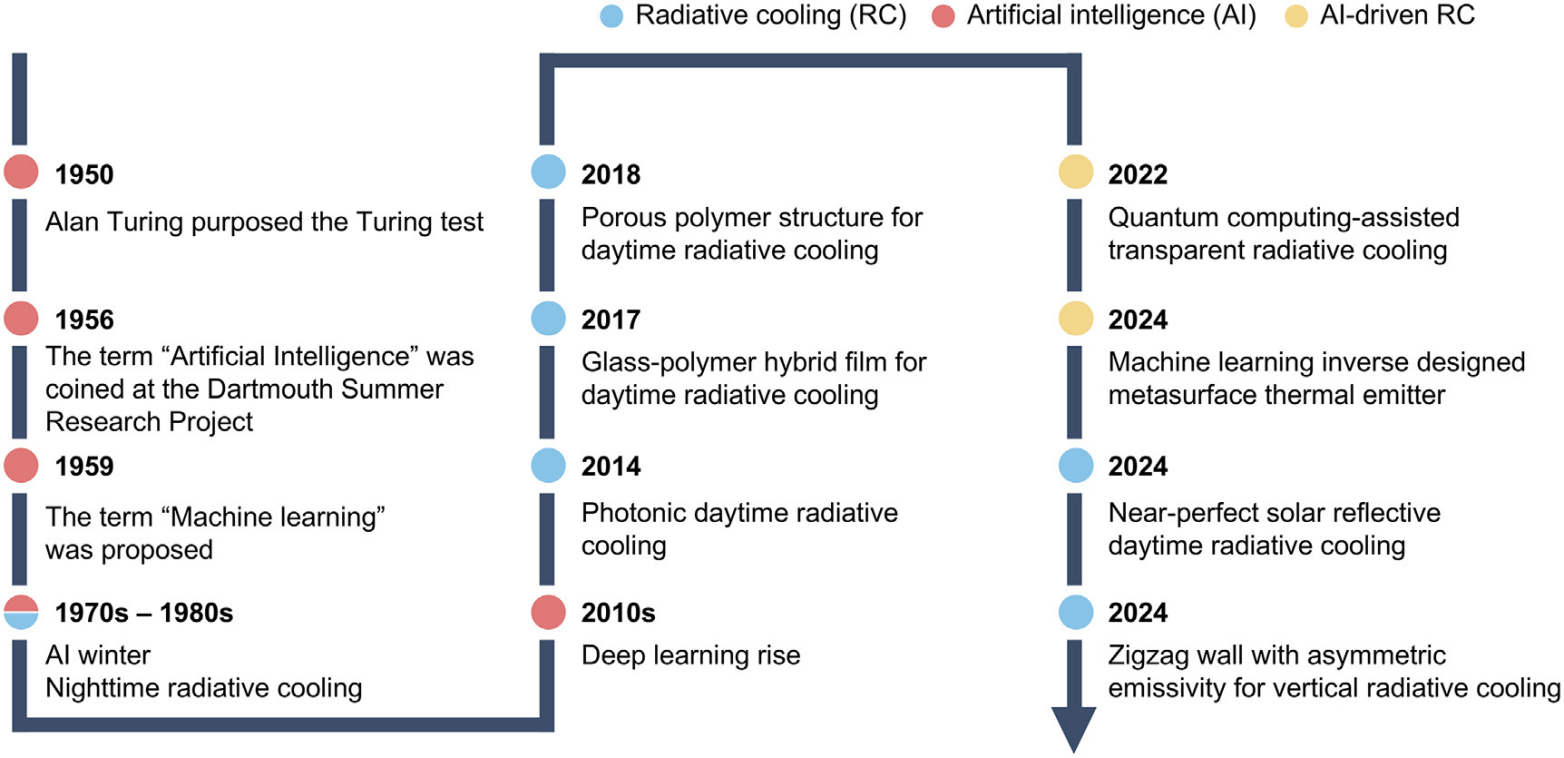
Timeline of key milestones in the evolution of artificial intelligence (red dots), radiative cooling (blue dots), and AI-driven RC technology (yellow dots)

In this regard, passive radiative cooling (PRC) technology has gained significant attention in recent decades due to its potential to address the global issues. The PRC is a sustainable cooling strategy that facilitates heat dissipation by emitting thermal radiation toward the extremely cold Universe (∼3 K), which serves as a natural infinite heatsink.^6^^,^^7^^,^^8^^,^^9^ Since the initial development of nighttime radiative cooling in the 1970s and 1980s, daytime radiative cooling systems have emerged leveraging nano-scale photonic structures to elaborately control the optical properties, thereby achieving sub-ambient cooling performance under direct solar irradiation (Figure 1).^8^^,^^10^^,^^11^^,^^12^^,^^13^^,^^14^^,^^15^^,^^16^^,^^17^^,^^18^^,^^19^^,^^20^^,^^21^^,^^22^ While sophisticated architectures based on photonic structures have been extensively designed for radiative cooling in the early stage, their high cost, complex manufacturing processes, and susceptibility to mechanical stress present substantial challenges.^23^

As the field of radiative cooling technology has progressed, polymer-based structures have been proposed as scalable, straightforward, and cost-effective alternatives, including polymer-metal hybrid films, porous polymer structures, and multilayered metafabric structures.^14^^,^^15^^,^^24^ Among these, porous polymer structures, capable of reflecting the incoming sunlight by exploiting the refractive index contrast between air and the polymer, have been significantly studied using various approaches such as phase inversion method,^14^ electrospinning technology,^25^ and template-casting method.^26^ However, polymer degradation issues, such as yellowing when exposed to ambient conditions, persist.^27^ Inorganic radiative cooling systems, which stably maintain the radiative cooling performance even under harsh conditions, have also emerged as a promising solution to address these challenges.^27^^,^^28^

Extensive research has been undertaken to develop radiative cooling technologies tailored to specific functionalities, addressing the various demands of practical implementation. Fundamentally, general radiative cooling (GRC) films reflect incoming solar radiation and emit thermal radiation.^29^^,^^30^^,^^31^ These GRC films, often exhibiting glossy or opaque white appearance, can achieve the sub-ambient cooling performance.^32^^,^^33^ Beyond this basic approach, several technologies are further being explored, including transparent radiative cooling (TRC), which transmits visible light while selectively controlling optical properties at other wavelengths.^34^^,^^35^^,^^36^^,^^37^^,^^38^ To implement the diverse radiative cooling technologies tailored to operational requirements, the precise control of optical properties is crucial.

The traditional strategies, including intuition-based approaches and trial-and-error methods guided by empirical observations and manual parameter tuning, are not ideal. The integration of artificial intelligence (AI) into the field of radiative cooling offers potential for accelerating advancements due to the ability of AI to perform tasks that require human intelligence or surpass human capabilities.^39^ AI, which has evolved significantly since its inception by pioneers such as Alan Turing and John McCarthy,^40^ has progressed from the era of “AI Winter” to its current state (Figure 1). Today, AI encompasses a diverse range of subsets, including machine learning (ML), deep learning, robotics, and neural networks, which are frequently used in combination, significantly enhancing our ability to address and solve complex engineering challenges.

**Figure 2:**
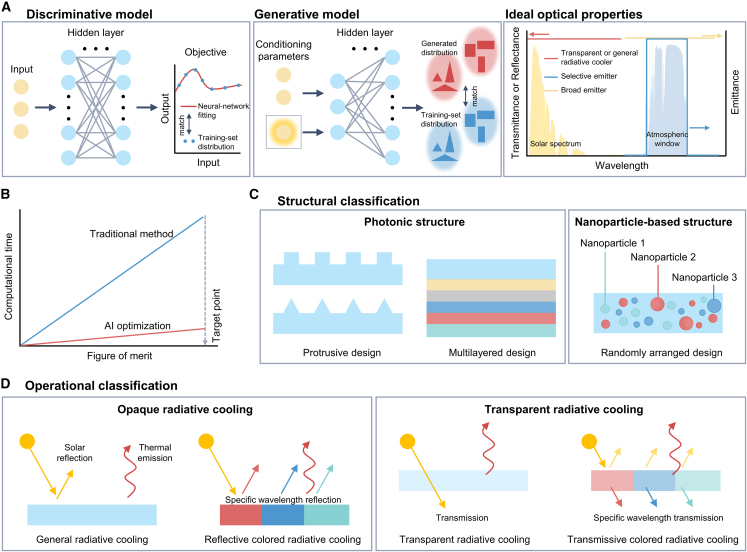
Radiative cooling technology with AI (A) Schematic illustrations of algorithm models, including discriminative and generative model, and optical properties for ideal radiative cooler. (B) Schemes showing comparison of computational times using AI optimization method and the traditional method. (C and D) Classification of AI-driven radiative cooling technology. (C) Structural classification. (D) Operational classification.

ML, a key subset of AI, enables systems to learn from data, discern underlying patterns, and tackle novel challenges. It involves steps such as data acquisition, feature engineering, model selection, and model evaluation.^41^ ML models are fundamentally categorized into discriminative models and generative models (Figure 2A). Discriminative models are designed to predict outcomes based on input data by distinguishing between different classes or categories.^42^ These models focus on identifying the decision boundaries that separate distinct classes. Specifically, these models help determine how different combinations of material composition, layer thickness, and processing conditions affect key performance indicators such as emissivity and reflectivity. It could also classify materials into categories such as high or low emissivity based on their structural properties. As such, the discriminative models serve to narrow down the most promising fabrication parameters by accurately predicting the resulting performance. Conversely, generative models are used for tasks such as data augmentation and simulation, generating new data samples that resemble the training data. By learning the underlying relationships in the material property space, these models can suggest new material configurations that exhibit desirable radiative cooling properties. This generative approach allows researchers to go beyond the limitations of available experimental data and explore innovative solutions in material design. Based on these approaches, the application of ML techniques in radiative cooling technology represents an emerging and rapidly advancing field. By utilizing input datasets derived from experimental observations or simulation data obtained through Monte Carlo simulations or density functional theory, researchers can enhance the efficiency and effectiveness of radiative cooling solutions. Recent advancements in this domain have fostered innovative approaches to address complex thermal management challenges, paving the way for more sustainable and optimized thermal control systems.^43^^,^^44^^,^^45^

This article provides a thorough examination of the evolution of radiative cooling technology facilitated by AI, focusing on the challenges and extraordinary progress emerging from the innovative paradigm. Our objective is to propose advanced engineering strategies to address the critical global challenges, particularly environmental issues, that remain prevalent today.

## General radiative cooling through AI technology

A myriad of studies has reported the GRC technology that achieves the sustainable cooling functionality by reflecting the incoming solar radiation and emitting thermal radiation within the atmospheric window (AW, 8–13 μm) spectrum toward cold space. For GRC films with an opaque appearance to achieve sub-ambient cooling, they must demonstrate selective emissivity within the AW spectrum.^46^ Broadband coolers, which emit across the entire infrared (IR) spectrum, are effective at ambient or higher temperatures. However, their high emissivity outside the AW spectrum results in significant absorption of downward atmospheric radiation, thereby limiting their cooling performance. Conversely, coolers with selective emissivity, particularly low emissivity outside the AW spectrum, reduce overall thermal emission but minimize the absorption of atmospheric and surrounding radiation, thus enhancing sub-ambient cooling performance. Thus, broadband coolers are suited for scenarios involving temperatures at or above ambient levels, while selective coolers are optimized for sub-ambient cooling applications.

Building on these fundamental principles, recent advancements have increasingly integrated AI into the design of radiative cooling systems, which aims to enhance efficiency and optimize architectural solutions by addressing the challenge of managing the inherently broad and dispersive emission characteristics of photonic materials (Figure 2B). The complexity of modern photonic structures, which often involves multiple geometric configurations and various photonic resonances, necessitates sophisticated design strategies to achieve improved performance in radiative cooling applications (Figure 2C). Moreover, omnidirectional emissivity, the ability to emit thermal radiation in the broad angle, is an important factor to minimize the external heating load. Recent study has designed the hybrid metasurface-based thermal emitters with high selectivity and omnidirectionality using artificial neuron network inverse design.^20^ Specifically, finite difference time domain simulation calculated the structural parameters, followed by the training of the neural network to predict the thermal emission. Subsequently, the optimization process for the input structural parameters was conducted by a backpropagation model, thereby designing an ideal selective thermal emitter. The resulting metasurfaces, composed of alternating disk-shaped configurations of SiO_2_ and Si_3_N_4_ at the microscale, exhibited a high emissivity of 0.92 across the AW, a selectivity factor of 1.8, and an emission angle of up to 80°, where the excitation of multiple Mie resonances enhanced thermal emission in the IR spectrum. However, the use of an Ag reflector and a nanoporous polyethylene film to enhance solar reflectance introduced a limitation in this approach, as the study focused exclusively on optimizing the IR spectrum using AI, without extending the optimization to the solar spectrum.

Inspired by the shells of snails that endure hot and arid climates, a radiative cooling structure with optimized solar reflection has been engineered using calcium carbonate (calcite – CaCO₃), a key component of snail shells.^47^ The design incorporates a multilayer architecture, comprising alternating layers of calcite and air gaps. To achieve optimal performance, the configuration was refined using a genetic algorithm coupled with the transfer matrix method, focusing on three critical objective functions: maximizing solar reflectance, enhancing emissivity within the AW, and improving the radiative cooling figure-of-merit (FOM). The resulting coating exhibited a significant improvement of solar reflectance to about 99.8%. Moreover, the well-established finding that a distribution of nanoparticle sizes enhances the solar reflectance, thereby achieving superior radiative cooling performance compared to a single particle size, has been recently explored through the integration of the theoretical Monte Carlo simulations and ML technique.^48^ AI can leverage datasets generated through Monte Carlo simulations to develop predictive models that approximate the outcomes with reduced computational resources. Unlike traditional Monte Carlo simulations, which must be re-executed for each new set of conditions, once trained, AI models can quickly estimate performance metrics such as cooling efficiency under various scenarios. Based on the significant advantage of AI over solely Monte Carlo simulations, given the challenges of achieving precise single-particle sizes in practical fabrication procedures, this finding suggests that the primary advantage of particle size distribution lies in their ability to support efficient preparing process, which enables a broader range of particle sizes while preserving high performance, thereby enhancing the practicality and feasibility of large-scale production.

While early AI-driven radiative cooling technologies primarily focused on the optimized design of photonic structures to achieve superior cooling performance, the experimental fabrication of these structures presents considerable challenges, including high costs, time-consuming manufacturing processes, and the complexity of fabrication techniques. To mitigate these limitations, there is a gradually increasing trend in research toward AI-driven radiative cooling technologies that move away from the dependence on photonic structures, instead prioritizing more facile and scalable fabrication processes.^49^ These developments are expected to surpass the limitations of traditional photonic structures, thereby facilitating the more widespread adoption and rapid advancement of radiative cooling techniques in real-world applications.

## Transparent radiative cooling through AI technology

Windows in buildings and vehicles contribute significantly to energy loss. To mitigate this issue, initial research has been conducted on energy-saving windows, such as low-emissivity glass windows that offer high visible transparency and high IR reflectivity. These windows effectively suppress radiative heat transfer and enhance thermal insulation. However, in warm climates, they can inadvertently cause an unexpected rise in indoor temperature by hindering radiative heat transfer for achieving cooling.

To address this challenge, early studies have focused on TRC technology. Initially, the TRC materials are overall transparent across the solar spectrum, while exhibiting high emissivity in the IR region (Figure 2D).^34^^,^^50^ Fundamentally, it is noticeable that TRC requires broadband thermal emission when compared to the opaque radiative cooling, as the temperature of the cooler surface can be comparable to or even exceed the ambient temperature in window applications. As window-based radiative cooling technology evolves for further maximizing the cooling performance, efforts have intensified to suppress the inward transmission of UV and near-IR (NIR) radiation while maintaining high transparency in the visible range.^51^^,^^52^^,^^53^ To achieve these goals, initial studies utilized theoretical calculations based on effective medium theory to develop transparent radiative cooling films. These films typically consist of alternating multilayers of Ag and SiO₂, which block solar IR energy (>89%), emit thermal radiation (>95%), and transmit visible light (>60%).^54^ However, these studies primarily focus on engineering photonic structures through theoretical calculations, which, while systemically engineered, present limitations in terms of efficiency and practicality.

Deep reinforcement learning method can offer an efficient approach for optimizing the TRC structures, surpassing traditional empirical and manual parameter tuning techniques.^55^ Specifically, among six candidate dielectric materials with negligible extinction coefficients in the solar spectrum, the optimization process through a value-based deep Q-Network involves selecting two materials and arranging them in an alternating stack to form a five-layer structure. Furthermore, another research has also explored the use of quantum computing to advance transparent radiative cooling technologies, promising significant improvements in both efficiency and practical implementation.^21^ Quantum annealing (QA) has been employed to optimize the design of TRC systems. QA employs quantum mechanism to tunnel through the objective function, potentially finding the global value more efficiently. In this research, alternating multilayers of SiO_2_, Si_3_N_4_, Al_2_O_3_, and TiO_2_, which exhibit negligible absorption within the solar spectrum, were optimized using a QA-assisted active learning method, thereby aiming to fabricate a TRC system that closely approximates the ideal performance. Specifically, an FOM was established to evaluate the extent to which the designed TRC system aligns with the ideal TRC, serving as the objective function in the optimization process. By employing the transfer matrix method, the optimization process minimized the FOM, thereby refining the TRC design to better align with the ideal model.

Colored radiative cooling (CRC) is highly promising due to its aesthetic appeals and multifunctionality. While the opaque CRC technologies have been extensively explored using various approaches such as coating pigments, photoluminescent effect, and structural coloration, they face significant challenges in achieving outstanding cooling performance due to the absorbed light to decrease the cooling efficiency. Transmissive CRC (tCRC), capable of transmitting specific wavelength while reflecting the overall solar spectrum for more vivid coloration, presents a viable solution to address the issues of opaque CRC films. Recent advancements in material informatics, particularly through the application of ML combined with experimental data, have shown great potential in accelerating the optimization of tCRC designs. By defining an FOM that emphasizes high transmittance within the desired wavelength range and negligible transmittance elsewhere, photonic structures have been optimized to achieve the global optimal performance. Specifically, the use of Bayesian algorithms, applied to sub-groups of candidate designs, has significantly expedited the optimization process, thereby achieving results approximately two orders of magnitude faster than traditional random search methods that evaluate all candidates in bulk.^56^ Each optimized tCRC design, containing red, green, and blue transmission, respectively, exhibited the outstanding sub-ambient radiative cooling performance. Nevertheless, even with advanced design strategies such as Bayesian optimization, genetic algorithms, and memetic algorithms, the process of iteratively refining intermediate designs is time intensive and typically restricted to achieving a single target color. As a result, if a different color is desired, the entire design process must be repeated from the beginning. To mitigate the challenging issues, inverse design method for developing transmissive coloration with radiative cooling has been reported.^57^ The tCRC film, composed of two operational components such as top selective emitter and bottom nanocavity structure for coloration, was optimally fabricated by employing a mixed-integer memetic algorithm for top selective emitter and then tandem neural network-assisted inverse design approach for bottom structure, respectively.

To date, research efforts aimed at realizing transparent radiative cooling technologies through the integration of AI techniques have primarily focused on optimizing the optical properties within the solar spectrum, particularly by blocking UV and NIR light while transmitting visible light. These studies are still in the early stages of development, with various AI methodologies being employed to enhance the performance of the transparent cooling technologies. Recently, however, there has been a shift toward exploring transparent radiative cooling systems with more specialized objectives. For instance, energy-saving windows, which realize multifunctional operation such as radiative cooling, energy harvesting, and heater in only one-structured device, have been reported.^58^ Additionally, haze radiative cooling technology, which also utilizes photonic structures, has been investigated to diffuse incoming light (73%), thereby improving indoor comfort and providing privacy protection.^59^ Another notable example is the development of photosynthetically active radiative cooling technologies.^60^ These systems, which also incorporate alternating multilayer photonic structures, are designed to promote plant growth by selectively transmitting wavelengths that are conducive to photosynthesis. As the field of transparent radiative cooling continues to expand with increasingly diverse applications, the more active integration of AI could significantly enhance the optimization of these technologies, leading to more efficient and robust solutions.

## Recommendations

Recent advancements in AI-based radiative cooling research have focused on refining the design of radiative coolers through the precise modulation of optical properties across both the solar and IR spectra. Although early-stage AI-based radiative cooling research, which remains in its nascent phase, has predominantly concentrated on optimizing photonic structures, recent studies have introduced alternative approaches. These newer methods leverage AI technologies to optimize nanoparticle sizes, addressing some of the limitations inherent in photonic structure-based techniques. This shift toward nanoparticle size distribution and volume ratio optimization holds promise for overcoming the challenges associated with photonic structures and offers potential advantages in terms of simpler fabrication processes and solution-based applications.

In line with the ongoing shift toward more practical methodologies, the development of AI-based radiative cooling technology should also be increasingly directed toward the integration of diverse manufacturing processes. The use of electrospinning technology into the radiative cooling has gained extensive attention due to its capacity for precise control over fiber morphology based on diverse processing parameters.^61^^,^^62^ Concurrently, ceramic-based materials for practical radiative cooling have been recently explored,^63^ offering distinct advantages such as exceptional thermal stability and durability even under the extremely high temperature conditions.^16^ The unique properties of ceramics present opportunities for enhancing the performance and longevity of radiative cooling systems. Likewise, research efforts are increasingly focusing on implementing radiative cooling technologies that extend beyond traditional photonic structures, utilizing diverse materials and architectures to achieve passive cooling functionality suitable for practical application. In this regard, we believe that employing AI technology for optimizing various radiative cooling structures has the potential to not only facilitate the efficient mass production of radiative cooling materials, enabling a smooth transition from lab-scale production to large-scale industrial applications, but also support the development of on-demand radiative cooling solutions tailored to specific applications.

**Figure 3:**
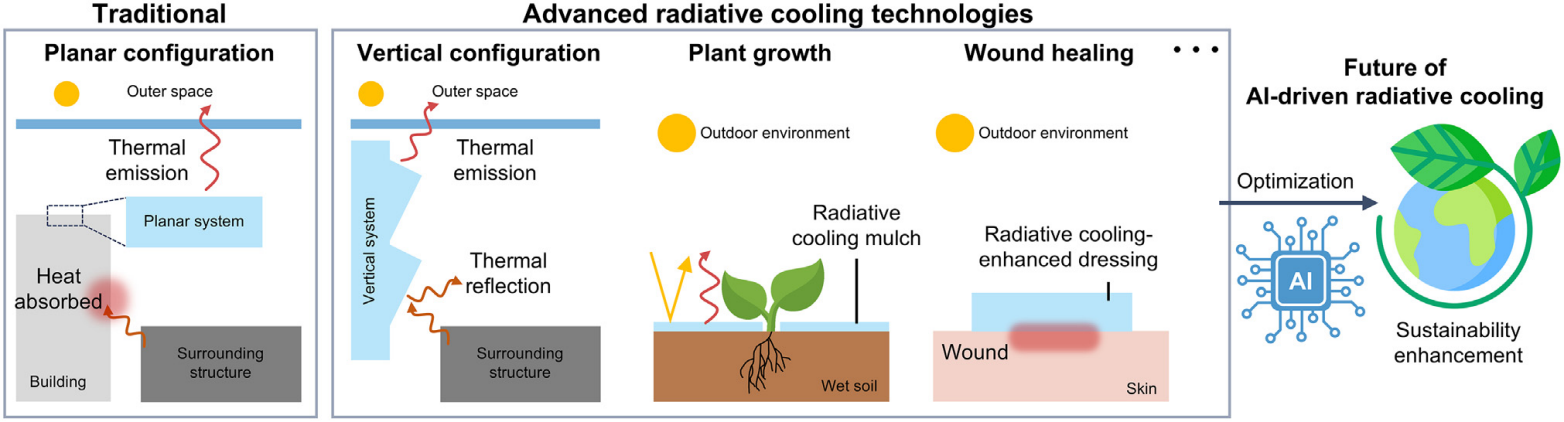
Future of AI-driven radiative cooling technology for significantly enhancing sustainability

Most recently, the application of radiative cooling technologies to vertical structures, particularly building walls, has emerged as a significant area of interest (Figure 3).^17^^,^^64^^,^^65^ The integration of AI into this domain must be critical, as the heat transfer mechanisms in vertical systems differ markedly from those in planar systems that have already been researched to date. Vertical configurations are prone to absorbing the radiated heat from the surrounding buildings and surfaces, particularly in urban environments, when compared to the planar systems that directly face upward to the sky. Consequently, a thorough understanding of the heat transfer mechanisms between vertical architecture and the surrounding environment, including factors such as view factor and orientation-specific heat transfer dynamics, is essential. With this understanding, AI-driven optimization of structural design could help achieve the ideal optical properties required for vertical configurations. Although the specific field of vertical radiative cooling is still in its nascent stages, we believe that such advancements will lead to more refined and effective radiative cooling technologies, addressing the overall building surfaces such as walls and rooftops, and ultimately contributing to global challenges like carbon neutrality.

Simultaneously, in recent years, the scope of radiative cooling research has expanded to address application needs such as agricultural mulching,^66^^,^^67^ enhancing photosynthesis,^59^^,^^60^ and wound healing.^68^ For instance, in agricultural applications, AI can help identify the optimal material composition, layering structures, and manufacturing parameters that maximize radiative cooling performance while ensuring durability and cost-effectiveness, thereby enhancing crop yield, minimizing water evaporation, and extending the lifespan of the mulch materials across various environmental conditions. Similarly, in the context of wound healing, AI can be employed to optimize radiative cooling materials to balance skin compatibility while avoiding patient discomfort, ensuring that the final product is not only efficient in delivering radiative cooling but also safe for direct application to sensitive skin areas. Despite significant advancements in radiative cooling technologies, AI integration has predominantly focused on conventional areas like buildings and vehicles. The application of AI to these newly emerging areas remains relatively underdeveloped. It is evident that AI-driven radiative cooling studies have not yet fully kept pace with the broader advancements in the field. Moving forward, leveraging AI technology to develop efficient radiative cooling solutions tailored to these advanced applications will be crucial, thereby not only broadening the scope of research and application in this field but also contributing to improving the quality of life.

## Acknowledgments

The work was supported by SNU-Global Excellence Research Center establishment project and the 10.13039/501100003725National Research Foundation of Korea Grant (RS-2024-00416938).

## Author contributions

Y.J. and S.H.K. led the drafting and editing of the manuscript.

## Declaration of interests

The authors declare no competing interests.
